# Research of storable and ready-to-use artificial red blood cells (hemoglobin vesicles) for emergency medicine and other clinical applications

**DOI:** 10.3389/fmedt.2022.1048951

**Published:** 2022-12-23

**Authors:** Hiromi Sakai, Tomoko Kure, Kazuaki Taguchi, Hiroshi Azuma

**Affiliations:** ^1^ Department of Chemistry, Nara Medical University, Kashihara, Japan; ^2^ Faculty of Pharmacy, Keio University, Tokyo, Japan; ^3^ Department of Pediatrics, Asahikawa Medical University, Asahikawa, Japan

**Keywords:** artificial oxygen carriers, blood substitutes, translational research, encapsulation, liposome, carbonylhemoglobin, methemoglobin (metHb)

## Abstract

Hemoglobin (Hb) is the most abundant protein in blood, with concentration of about 12–15 g/dl. The highly concentrated Hb solution (35 g/dl) is compartmentalized in red blood cells (RBCs). Once Hb is released from RBCs by hemolysis during blood circulation, it induces renal and cardiovascular toxicities. To date, hemoglobin-based oxygen carriers of various types have been developed as blood substitutes to mitigate the Hb toxicities. One method is Hb encapsulation in phospholipid vesicles (liposomes). Although the Hb toxicity can be shielded, it is equally important to ensure the biocompatibility of the liposomal membrane. We have developed Hb-vesicles (HbV). A new encapsulation method using a rotation-revolution mixer which enabled efficient production of HbV with a high yield has considerably facilitated R&D of HbV. Along with our academic consortium, we have studied the preclinical safety and efficacy of HbV extensively as a transfusion alternative, and finally conducted a phase I clinical trial. Moreover, carbonyl-HbV and met-HbV are developed respectively for an anti-inflammatory and anti-oxidative agent and an antidote for poisons. This review paper specifically presents past trials of liposome encapsulated Hb, biocompatible lipid bilayer membranes, and efficient HbV preparation methods, in addition to potential clinical applications of HbV based on results of our *in vivo* studies.

## Introduction

Blood donation and transfusion are routinely practiced for sustaining human health and welfare. Their history informs us of the important endeavors of researchers and clinicians at establishing present modes of safer blood transfusion, and we have to continue our endeavors to challenge the remaining difficulties. Screening of the donated blood for hepatitis viruses B, C and E, HIV, west Nile virus etc. by nucleic acid amplification testing (NAT) has mostly eliminated transfusion-related infections. Nevertheless, emergent infectious viruses threaten humanity continuously. Concentrates of donated red blood cells (RBCs) can be stored in a refrigerator for 6 weeks in the US and EU, but for only 3 weeks in Japan. Rare blood-type RBCs are frozen with a cryoprotectant for long-term storage, but the cryoprotectant must be removed before infusion. Such limited storage conditions impose burdens on logistics, especially for remote islands and rural areas and stockpiling for emergency needs. Crossmatching and blood-type testing immediately before urgent transfusion are particularly time-consuming for life-saving practices. Even in economically developed countries, clinicians experience urgent situations in which blood transfusion is not available to treat patients. These difficulties have spurred us to develop artificial red cells that can eventually be substituted for RBC transfusion where blood transfusion is not available (1). Worldwide deaths from hemorrhage are estimated as 1.9 million per year, with 1.5 million resulting from physical trauma (2).

Hemoglobin (Mw. 64,500) is the most abundant protein in blood. About two million Hb molecules are compartmentalized in a single red blood cell (RBC). The intracellular Hb concentration, which is as high as 35 g/dl, makes the Hb concentration of blood as high as 12–15 g/dl. Because blood type antigens are present on the outer surface of RBCs, an early idea was to use purified Hb as an oxygen-carrying fluid that is free of any blood type. Nevertheless, that effort was unsuccessful because of various toxic effects. In spite of its abundance in blood, Hb becomes toxic when it is released from RBCs. Dissociation of tetramer Hb subunits into two dimers occurs, which proceeds to induce renal toxicity. Entrapment of a gaseous messenger molecule, NO, induces vasoconstriction, hypertension, neurological disturbances, and malfunction of esophageal motor functions (3–6). An aqueous solution of chemically modified Hb-based oxygen carriers (HBOCs) exhibits a colloid osmotic pressure that sometimes exceeds the physiological value (20–25 mmHg) in spite of its low Hb content, thereby having a potential to cause volume overload (7). Some first generation chemically modified HBOCs presented side effects in clinical trials: higher risks of myocardial infarction and death (8, 9). These side effects of molecular Hb imply the importance of the cellular structure and larger particle size of HBOCs (10).

## Encapsulation of hemoglobin into liposomes to mimic red blood cells

### Concept of hemoglobin encapsulation in liposomes

Pioneering Hb microencapsulation work was first performed by Chang in 1957 (11) using a polymer membrane. Japanese groups followed his attempt to test Hb encapsulation with gelatin, gum Arabic, silicone, etc. (12). Nevertheless, regulating the particle size to be appropriate for blood flow in the capillaries and obtaining sufficient biocompatibility were extremely difficult. Bangham and Horne reported in 1964 that phospholipids assemble to form vesicles in water (13), suggesting that the vesicles (liposomes) can encapsulate water-soluble functional materials in their inner phase (14). The lipid membrane of liposomes somewhat resembles a biomembrane. It should be more biocompatible than a synthetic polymer membrane. Djordjevici and Miller in 1977 first reported a liposome-encapsulated Hb (LEH) as an artificial oxygen carrier (15). Following their trial, many laboratories tested Hb encapsulation using liposomes with various lipid compositions and preparation methods (Table 1) (16–44). The addition of cholesterol to phospholipid is a standard method of stabilizing the packing of the lipid membrane and of reducing its curvature to produce larger liposomes. Inclusion of a negatively charged lipid is also a standard recipe to provide negative charges on the liposomal surface, which is effective to reduce the lamellarity of liposomes and to increase the volume of inner aqueous phase, thereby leading to higher Hb encapsulation efficiency (33, 45). Exceptionally, the trials of entry Nos. 3, 5, 16, 21, and 22 did not use negatively charged lipids (18, 20, 42, 43). It is noteworthy that not only liposomes but also polymersomes and other submicrometer capsules made of synthetic biodegradable polymers are tested extensively for Hb encapsulation (46–48). Detailed results of their safety and efficacy studies are awaited.

**Table 1:** Trials of liposome encapsulated Hb with various lipid compositions and preparation methods.

No.	Lipid composition for Hb encapsulation	Points of preparation method	References (Institution)
**1970s–1980s**			
1	L-α-phosphatidylcholine/cholesterol/palmitic acid	Sonication	(15) (Univ. of Illinois)
2	EYL/cholesterol/bovine brain PS DSPC, DPPC, or DMPC/cholesterol/dicetylphosphate or DMPG	Extrusion	(16, 17) (Naval Res. Lab.)
3	EYL/carboxymethyl chitin	Reverse phase evaporation	(18) (Sci. Univ. Tokyo)
4	EYL/cholesterol/DPPA/α-tocopherol	Reverse phase evaporation, Extrusion	(19) (Univ. California, San Francisco)
5	Diacetylene phospholipid/cholesterol UV polymerization	HbCO, Sonication	(20) (State Univ. N.Y.)
6	HSPC/cholesterol/dicetylphosphate or DMPG (Trehalose is added as a lyoprotectant.)	Microfluidizer	(21, 22) (Naval Res. Lab.)
7	EYL/cholesterol/PS/PA/α-tocopherol	Detergent dialysis	(23) (Univ. Tübingen)
**1990s**			
8	DSPC/cholesterol/DMPG/α-tocopherol (Trehalose is added as a lyoprotectant.)	Bovine Hb, Thin film hydration and emulsification	(24) (Naval Res. Lab.)
9	HSPC/cholesterol/myristic acid/α-tocopherol/DPPE-PEG	Microfluidizer	(25, 26) (Terumo Corp.)
10	HSPC/DMPG/α-tocopherol/carboxymethyl chitin	Reverse phase evaporation	(27) (McGill Univ.)
11	DODPC/cholesterol/octadecadienoic acid gamma-ray polymerization	HbCO, Extrusion	(28–30) (NOF Corp.)
12	EYL/cholesterol /dicetylphosphate/α-tocopherol	Freeze–thaw method	(31) (Univ. Pennsylvania)
13	DPPC/cholesterol/DPPG or palmitic acid	HbCO, Extrusion	(32, 33) (Waseda Univ.)
14	DPPC/cholesterol/DPPG/DSPE-PEG_5000_	HbCO, Extrusion	(34) (Waseda Univ.)
15	EYL/cholesterol/α-tocopherol/eggPA	Reverse phase evaporation	(35) (Chung-Yuan Christian Univ.)
16	DSPC/cholesterol/DSPE-PEG_5000_/α-tocopherol	αα-crosslinked human Hb, Microfluidizer	(36) (Univ. Texas San Antonio)
**2000s**			
17	DPPC/cholesterol/DHSG/DSPE-PEG_5000_	HbCO, Extrusion	(37, 38) (Waseda Univ.)
18	DMPC/cholesterol/DMPG/DSPE-PEG_2000_/actin	Extrusion	(39) (Univ. Notre Dame)
19	HSPC/cholesterol/stearic acid/DSPE-PEG_5000_	Lipid paste rapid dispersion	(40) (Terumo Corp.)
**2010s–2020s**			
20	DPPC/cholesterol/HDAS/α-tocopherol/HDAS-PEG_2000_	High pressure homogenization	(41) (Univ. Notre Dame)
21	EYL/cholesterol/DPSE-PEG_2000_	Thin film hydration and sonication	(42) (Zhejiang Univ.)
22	DSPC/cholesterol/DSPE-PEG_5000_	HbCO, cell disruptor	(43) (Ohio State Univ.)
23	DPPC/cholesterol/DHSG/DSPE-PEG_5000_	HbCO, Rotation-revolution mixer for encapsulation, Decarbonylation and deoxygenation for a long-term storage	(44) (Nara Med. Univ.)

**Abbreviations**: EYL, egg yolk lecithin; PS, phosphatidylserine; DSPC, 1,2-distearoyl-*sn*-glycero-3-phosphatidylcholine; DPPC, 1,2-dipalmitoyl-*sn*-glycero-3-phosphatidylcholine; DMPC, 1,2-dimyristoyl-*sn*-glycero-3-phosphatidylcholine; DMPG: 1,2-dimyristoyl-*sn*-glycero-3-phosphatidylglycerol; DPPA, 1,2-dipalmitoyl-*sn*-glycero-3-phosphatidic acid; HSPC, hydrogenated soy phosphatidylcholine; DODPC, 1,2-dioctadecadienoyl-*sn*-glycero-3-phosphatidylcholine; DPPE, 1,2-dipalmitoyl-*sn*-glycero-3-phosphatidylethanolamine; DSPE, 1,2-distearoyl-*sn*-glycero-3-phosphatidylethanolamine; DHSG, 1,5-*O*-dihexadecyl-*N*-succinyl-L-glutamate; HDAS, hexadecyl-carbamoyl-methyl-hexadecanoate; HbCO, carbonylhemoglobin.

### Optimal lipid compositions for stability and safety of liposome encapsulated hemoglobin

**Figure 1:**
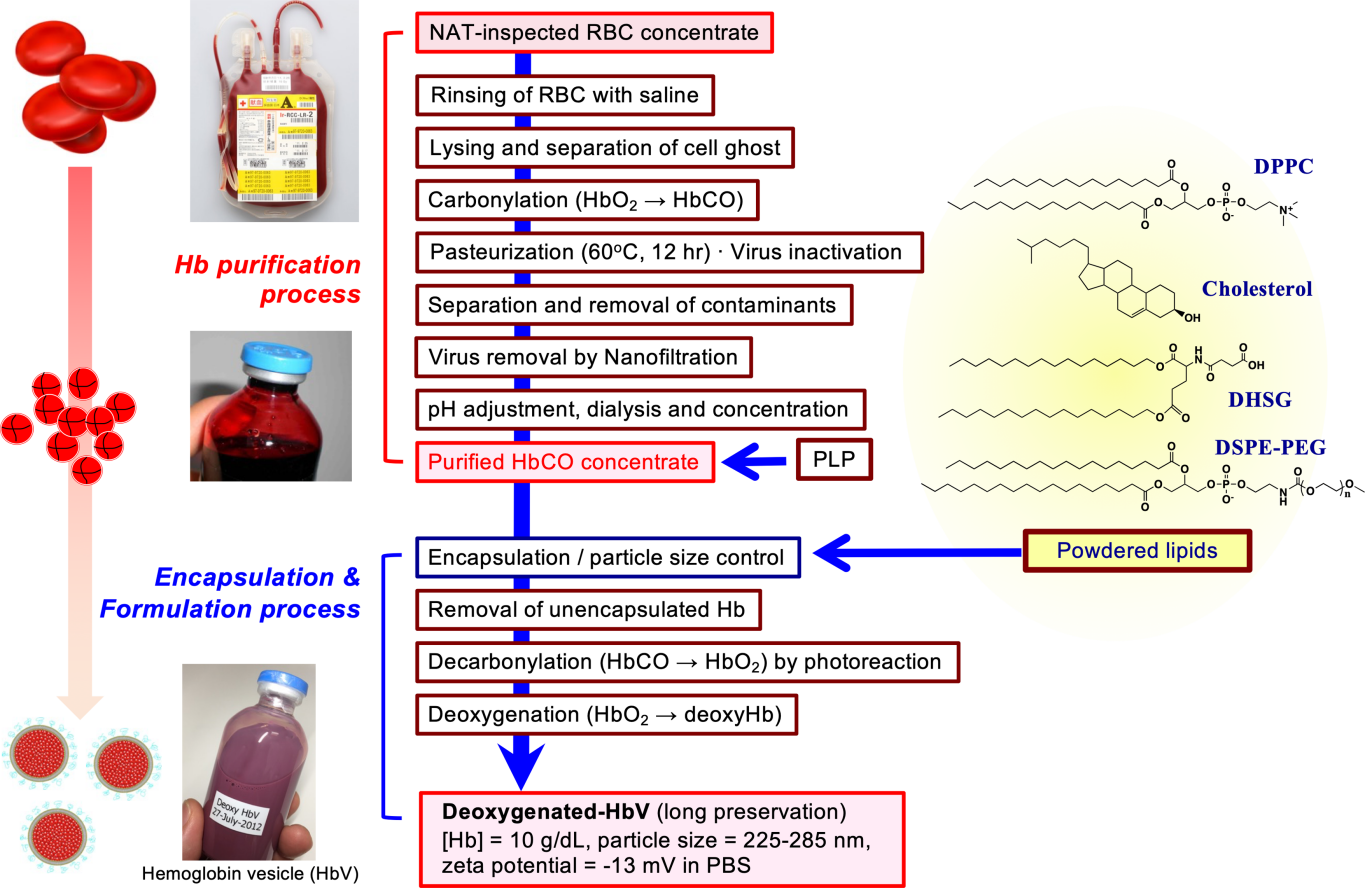
Outline of production scheme of HbV. HbCO solution was purified from nucleic acid amplification test (NAT)-inspected human red blood cells concentrate obtained from Japanese Red Cross Kinki Block Blood Center. Cells were rinsed with saline and lysed by gentle osmotic shock. The erythrocyte ghost was separated by ultrafiltration. After carbonylation, the resulting HbCO was pasteurized at 60 °C for 12 h, nano-filtrated, dialyzed, passed through anion-exchanging resins, and ultrafiltered to concentrate to 40 g/dl. Pyridoxal 5′-phosphate (PLP) was added to HbCO at the molar ratio of 1.0. The powdered lipids comprised of 1,2-dipalmitoyl-*sn*-glycero-3-phosphatidylcholine (DPPC), cholesterol, 1,5-*O*-dihexadecyl-*N*-succinyl-L-glutamate (DHSG), and 1,2-distearoyl-*sn*-glycero-3-phosphatidylethanolamine-*N*-poly(ethylene glycol) (PEG_5000_, DSPE-PEG) at a molar ratio of 5:4:0.9:0.03. The HbCO encapsulation and particle size control were performed using the kneading method with a rotation–revolution mixer (44). The unencapsulated Hb was removed by ultrafiltration; HbCO in the vesicles suspended in saline solution was converted to oxyhemoglobin by photoillumination and oxygen flow. The suspension was deoxygenated completely, purged in plastic bags, and sealed with an oxygen adsorber in aluminum bags for long-term storage at 2–8 °C.

Liposome is categorized as a molecular assembly of lipids formed through hydrophobic interaction among lipids. It is generally regarded as an unstable and fragile capsule, especially when using unsaturated phospholipids such as egg yolk lecithin. To stabilize LEH for long-term storage, polymerizable phospholipids that included diacetylene or diene groups in the phospholipid molecules (entry Nos. 5 & 11) were once tested (5, 28–30). After Hb encapsulation, the lipid was polymerized by UV irradiation or gamma-ray irradiation. In the case of diene-containing phospholipid, the obtained gamma-ray irradiated LEH was so stable that it was able to be frozen and thawed or freeze-dried and rehydrated without structural damage (49). However, one difficulty was clarified from animal experiments: the polymerized liposome could not be metabolized in the reticuloendothelial system. It remained in the liver and spleen for a long time (30). Other trials include surface coverage with polymer chains, such as carboxymethyl chitin (entry No. 3) and actin (entry No. 18) (18, 39). Rudolph et al. (23) tested freeze-drying and rehydration procedures of LEH in the presence of trehalose as a cryoprotectant as well as a lyoprotectant (entry No. 7). Since Yoshioka et al. reported PEGylation of LEH in 1989, it has become a standard method to stabilize the dispersion state during storage and during blood circulation (Entry No. 9) (50). In our research of so-called Hb-vesicles (HbV), we confirmed that deoxygenated HbV can be stored for over two years at room temperature using a saturated phospholipid, DPPC, to avoid lipid peroxidation and the combination of PEGylation (44) (Figure 1). PEGylation of liposomes is generally intended to prolong their circulation half-lives by the stealth effect (51). An additional benefit of PEGylation is to improve the dispersion state of liposomes during storage and in blood plasma or in plasma expanders (52, 53).

The toxicities of Hb can be shielded by liposomal encapsulation to mimic the physiological structure of erythrocytes. However, another important point is whether the liposomal lipid membrane is sufficiently biocompatible. The surface property of liposome is an important factor to assess safety. Reducing interactions with negatively charged plasma proteins and vascular endothelial cells is important. As shown in Table 1, many laboratories use negatively charged phospholipid components such as phosphatidic acid (PA) (19), phosphatidyl serine (PS) (16, 17), phosphatidyl glycerol (PG), and fatty acids (32–34). Later, it was clarified that such negatively charged lipids induce complement activation or platelet activation (40, 54–58). In our case, we use DHSG as a negatively charged synthetic lipid for HbV, which does not induce complement activation or platelet activation in preclinical animal experiments (37, 38, 44, 56–58) (Figure 1). Numerous selections of lipid species and compositions are present to make liposomes. Fortunately, the present lipid compositions of HbV: DPPC/cholesterol/DHSG/DSPE-PEG_5000_ show sufficient stability and biocompatibility.

### Preparation methods of liposome encapsulated hemoglobin

Because liposomal drugs for cancer and antifungal therapies are approved and because liposomes are used experimentally as a model of biomembrane, various preparation methods have been reported (14). Traditional methods include (i) sonication, (ii) reverse phase-evaporation method using an organic solvent such as diethyl ether, ethanol, or tertial butanol, and (iii) solubilization with detergent following dialysis. However, these methods would not be appropriate for Hb encapsulation for *in vivo* use because of the possibility of denaturation of Hb as a protein and incomplete removal of an organic solvent or a detergent. Another method is (iv) extrusion (32, 38). One parameter of LEH to express the oxygen-carrying capacity is the weight ratio of Hb to total lipid: Hb/Lipid. This parameter takes in increasingly larger value with encapsulation of larger amounts of Hb in liposomes with a smaller amount of lipids. Accordingly, the concentration of Hb for encapsulation is expected to be as high as 35–45 g/dl, which is similar to the corpuscular Hb concentration in erythrocytes (35 g/dl). Such a concentrated Hb solution becomes exponentially viscous (50 cP or more) (44, 59). For encapsulation, it must be mixed with lipids (thin lipid films prepared on the inner surface of flask, or freeze-dried lipid powder) (38). The resulting highly viscous mixture is extruded through membrane filters to regulate the particle size. Here the difficulty is clogging of the filter because of the large amount of Hb and lipids. To prevent filter clogging, the amount of lipids had to be reduced to about 6 g/dl, resulting in insufficient encapsulation efficiency to only about 20%. (v) Microfluidizer was often used to reduce the liposome size by a head-on collision of fast fluid flows (21). However, the fluids require lower viscosity for the fast flow and the amount of lipid to be added to Hb is limited, resulting in lower encapsulation efficiency.

To overcome such difficulties in preparation, we developed (vi) a new kneading method with a rotation-revolution mixer (44). The Hb encapsulation efficiency was increased dramatically to about 70%, which is nearly the closest packing factor of 74%, because the kneading method enabled mixing of a highly concentrated carbonylhemoglobin (HbCO) solution (40 g/dl) and a considerably large amount of powdered lipids in only 10–20 min. The high viscosity of the Hb-lipid mixture paste (ca. 10^3^–10^5^ cP) favorably induces frictional heat by kneading and increases the paste temperature (ca. 60 °C), which facilitates lipid dispersion and liposome formation. During the kneading operation using a thermostable HbCO solution, Hb denaturation was prevented. After HbCO in HbV is converted to HbO_2_ by photolysis, HbO_2_ is converted to deoxyHb for the long-term storage of HbV. The kneading method is apparently the most suitable for the preparation of HbV made of a viscous paste of the Hb and lipids. Actually, it has enabled scaling up of HbV production and has facilitated preclinical and clinical studies in our project (60).

## Safety studies of hemoglobin vesicles

The volumes of blood donation are 200 or 400 ml in Japan. It means that a hemorrhage of 400 ml blood loss does not affect physiological performance to any considerable degree. Therefore, a situation in which a blood substitute is necessary is estimated at a massive hemorrhage of 1,000 ml or more. The Hb concentration of blood is 12–15 g/dl. Blood is a concentrated RBC dispersion suspended in an aqueous solution of plasma proteins and other various solutes. Blood has physiologically adjusted viscosity, and crystalloid and colloid osmotic pressures. A blood substitute should also possess a compatible oxygen-carrying capacity and Hb concentration. In the case of HbV, the Hb concentration is adjusted to 10 g/dl, which is slightly higher than the transfusion trigger: 6–7 g/dl. Not RBCs but plasma proteins show colloid osmotic pressure of whole blood. The HbV is suspended in a physiological saline solution and the suspension does not possess colloid osmotic pressure. Therefore, HbV infusion requires co-injection or addition of plasma expanders such as human serum albumin, hydroxyethyl starch, modified fluid gelatin, or dextran depending on the situation, to adjust the colloid osmotic pressure, as does RBC transfusion (53, 61). Unlike conventional liposomal drugs used for cancer therapy, the volume of HbV for injection should be large: 16 ml/kg or more. Because of the necessity for massive infusion for HbV with high Hb and lipid concentrations, its safety in preclinical stages has been scrutinized in terms of blood compatibility, immunological (62), hematological (57), and cardiovascular effects, vital organ function, biodistribution (63, 64), excretion, etc. No hemolysis of HbV occurs during blood circulation. However, HbV is finally phagocytosed by macrophages, especially in the spleen and liver (65), which causes the main side effect of transient hepatosplenomegaly in rodent models, although the HbV in macrophage phagosomes disappears completely in two weeks; it is excreted through urine and feces (64, 66). Hemosiderin deposition, which is often observed in patients receiving repeated blood transfusion, was confirmed after repeated infusion of HbV in rats (65). All the relevant toxicological data have been reported and summarized elsewhere (67). One important physicochemical characteristic of HbV is that the HbV particles do not form any sediment after conventional centrifugation and show interference effect of “hemolysis” and “lipidemia” on blood clinical chemistry, but they do in the presence of a high molecular weight dextran. This feature enables easy separation of HbV from blood plasma specimen and contributes to accurate analyses in blood clinical chemistry to examine organ function (52).

## Potential clinical applications of hemoglobin vesicles

### Hemoglobin vesicles as an oxygen carrier

The efficacy of storable and ready-to-use HbV as a transfusion alternative has been tested by many animal models in our academic consortium, as presented in Table 2 (34, 53, 68–116). The ultimate purpose for R&D of HbV is to use the HbV fluid as a transfusion alternative, especially in urgent situations when RBC transfusion is not available. In fact, HbV has demonstrated effectiveness as a resuscitative fluid in animal models of hemorrhagic shock (72–80), peri-operational or injured uncontrolled massive hemorrhage (81, 84–86), and obstetric hemorrhage (82, 83). Under such circumstances, the level of blood exchange should exceed 50%. Co-injection of a plasma expander is necessary for such extreme conditions to maintain colloid osmotic pressure and the resulting blood volume. Hagisawa et al. clarified that intraosseous infusion of HbV is possible because of its smaller particle size than RBC. It easily enters osseous blood vessels to whole body blood circulation (79). This feature is particularly beneficial when peripheral blood vessels are collapsed and inaccessible. Moreover, HbV can replace packed RBCs as a priming solution for an extracorporeal membrane oxygenator during cardiovascular surgery. As an oxygen carrier, HbV can also be used for oxygen therapeutics for local ischemic diseases, brain protection at apnea, oxygenation of tumors for sensitization (94), and as an ^15^O carrier for positron emission tomography (95, 96), a perfusate for transplant organs (97–100), and an oxygen carrying medium for cell culturing (101). As a target dye, HbV is useful for dye laser irradiation therapy of port-wine stains (102, 103).

**Table 2:** Potential clinical application of HbV, not only as an O_2_-carrier, but also a CO carrier and an antidote, evidenced by academic consortium.

State of HbV	Application	Test Animal Species	References
Deoxy-HbV* (O_2_-HbV)	Isovolemic hemodilution (repeated injection at hemorrhage)	Wistar rats, SD rats	(34, 53, 68, 69)
		Syrian golden hamsters	(70, 71)
	Hemorrhagic shock	Wistar rats, Lewis rats	(72–74)
		Jpn or NZ white rabbits	(75–79)
		Beagle dogs	(80)
	Uncontrolled hemorrhage	Wistar rats	(81)
	Obstetric hemorrhage	Jpn white rabbits	(82, 83)
	Perioperational transfusion at pneumonectomy	C57BL/6 mice	(84)
		Wistar rats	(85)
		Beagle dogs	(86)
	Pre-eclampsia	Wistar rats	(87)
	Priming of ECMO	Wistar rats	(88)
	Brain ischemia	Wistar rats	(89)
	Skin flap ischemia	Syrian golden hamsters	(90)
		DDY mice	(91)
	Ischemia-reperfusion injury of heart	Wistar rat heart	(92)
	Brain protection at apnea	SD rats	(93)
	Tumor	C57BL/6 mice	(94)
	^15^O-PET	SD rats	(95, 96)
	Organ perfusion	BALB/c mice intestine	(97)
		Wistar rat hind leg	(98)
		Cross-bred pig liver	(99, 100)
	Cell culturing	Rat hepatocyte	(101)
	Dye laser irradiation of port-wine stains	Chicken wattle	(102)
		Jpn white rabbits	(103)
CO-HbV	Hemorrhagic shock	Wistar rats	(104, 105)
	Pulmonary fibrosis	Sea-ICR mice	(106)
	Colitis	Sea-ICR mice	(107)
	Pancreatitis	Balb/cN mice, Sea-ICR mice	(108, 109)
	Dye laser irradiation of port-wine stains	Jpn white rabbits	(110)
	Cisplatin-induced acute kidney injury	ICR mice	(111)
	Tracheal transplantation	C57BL/6 mice	(112)
met-HbV	Cyanide poisoning antidote	ddY mice	(113, 114)
	Azide poisoning antidote	ddY mice	(115)
	Hydrogen sulfide poisoning antidote	ddY mice	(116)

**Abbreviations**: ECMO, extracorporeal membrane oxygenator; PET, positron emission tomograph.

* For oxygen delivery by O_2_-HbV, the agent is stored as Deoxy-HbV before infusion.

### Hemoglobin vesicles as a carbon monoxide carrier

**Figure 2:**
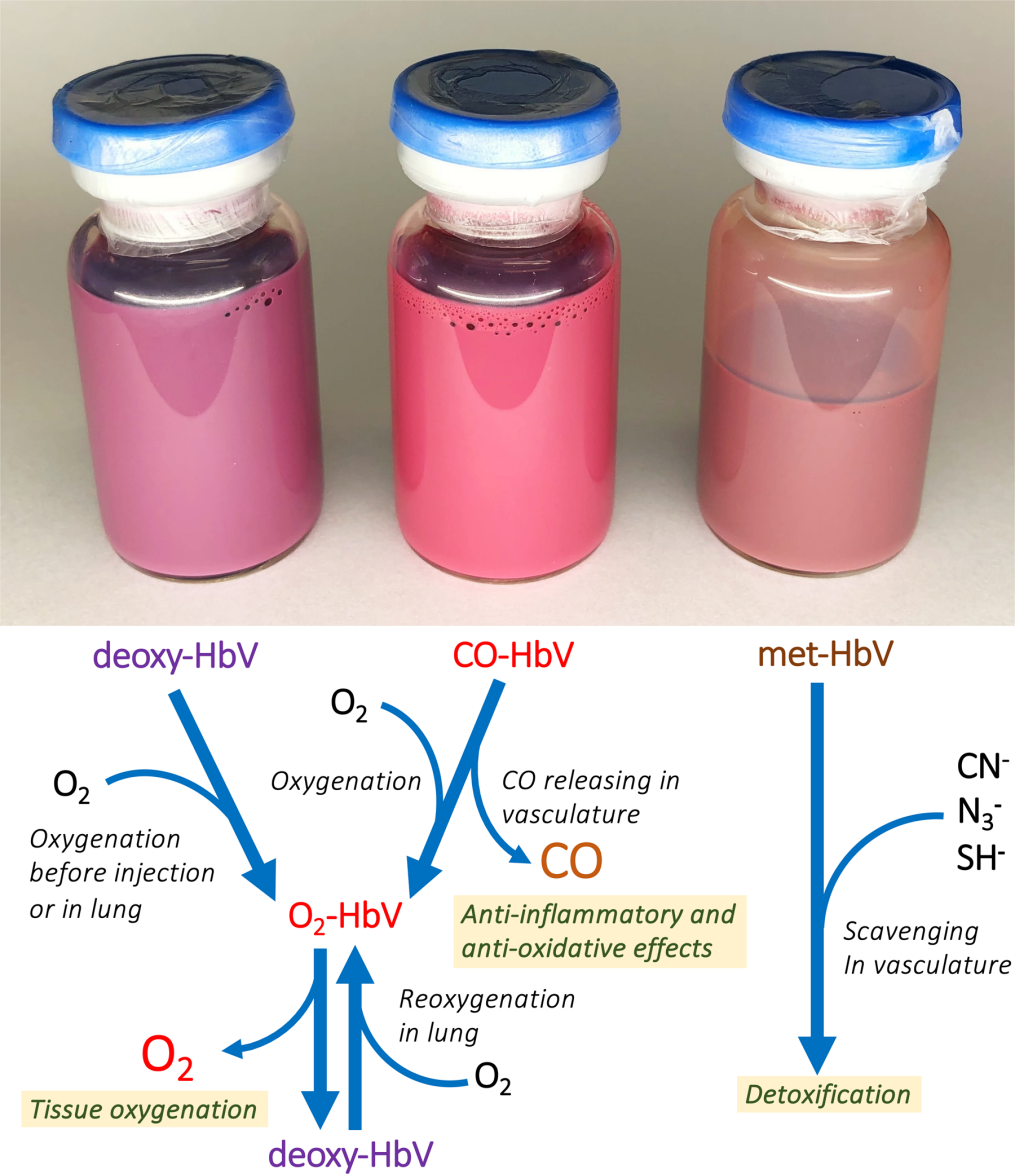
Glass vials containing deoxy-HbV, CO-HbV and met-HbV. The colors differ depending on the state of encapsulated Hb. Deoxy-HbV is for an O_2_-carrier, CO-HbV for a CO carrier, and metHbV for an antidote of poisonous materials. They can all be stored for years at room temperature.

Carbon monoxide has been tested as an anti-oxidative and anti-inflammatory agent. Direct inhalation of diluted CO gas has been reported, as have the intravenous infusion of CO-bound RBCs and various CO-releasing nanocarriers and molecules (117, 118). Studies have confirmed that CO-bound HbV (CO-HbV) is effective for resuscitation from hemorrhagic shock mitigating ischemia reperfusion injury (104, 105). Moreover, Nagao and Taguchi et al. (106–109) have clarified CO-HbV as a therapeutic agent for colitis, pancreatitis, and cisplatin-induced acute kidney injury (Table 2). After releasing CO, HbV becomes an O_2_ carrier (Figure 2). For all experiments, the target molecule of CO should be enzymatic heme proteins related to generation of reactive oxygen species (ROS), such as NADPH oxidase, and cytochrome C oxidase. CO should prevent the activities of such ROS-generating enzymes. CO is well known as a poisonous gas but here, poisoning of ROS is treated by another poison: CO. It is noteworthy that histopathological studies of rat brain have clarified that CO-HbV administration did not lead to marked hippocampal damage (105). Up to 32 ml/kg dosage of CO-HbV did not induce any behavioral abnormalities and the effect of CO-HbV on the central nervous system seems minimal (119).

### Hemoglobin vesicles as an antidote

HbO_2_ in HbV gradually autoxidizes and becomes metHb, thereby losing its oxygen-carrying capacity. Stoichiometric addition of sodium nitrate to HbO_2_ spontaneously converts it to metHb. Suzuki and Taguchi et al. (113–116) clarified that vesicles containing metHb (metHbV) are useful as an antidote for poisons such as cyanide, azide, and hydrogen sulfide, all of which bind strongly to metHb (Table 2, Figure 2). They confirmed the efficacies of metHbV as antidotes using rodent models. For example, the efficacy as antidotes for cyanide poisoning is higher than conventional treatments with hydroxocobalamin and nitrous acid compounds. Although such an agent would not be required frequently in normal times, it is expected to be indispensable for emergency medicine in some accident-related or disaster-affected situations.

## Phase 1 study of hemoglobin vesicles as a transfusion alternative

Many laboratories have attempted Hb encapsulation using liposomes, as shown in Table 1. Because of difficulty in ascertaining and developing optimal lipid compositions and efficient production methods, most groups eventually terminated their development. We have continued R&D of HbV and have confirmed their safety and efficacy through abundant preclinical studies. Our new kneading method to encapsulate Hb has facilitated their large-scale production and has supported our R&D projects overall. After full consultation with the Pharmaceuticals and Medical Devices Agency (PMDA), items of evaluations using rodents and dogs under GLP guideline were set in 2016. Subsequently, GLP non-clinical safety evaluations were conducted by private contracting laboratories and were completed in 2019 (60). From 2020, we started a small-scale GMP production of HbV at the Nara Medical University Cell Processing Center. Our academic consortium initiated a phase 1 (first-in-human) study of HbV in 2020 (Registration number, jRCT2011200004) (120) with the close assistance of clinical research organizations of Asahikawa Medical University Hospital Clinical Research Support Center and Hokkaido University Hospital Clinical Research and Medical Innovation Center.

The study examined three cohorts #1, #2, and #3 (*n *= 4), respectively, of 10, 50, and 100 ml dosage. Subjective symptoms, vital signs, electrocardiography, and hematological and biochemical parameters were observed. No severe adverse event was observed for any subject. No hypertension was observed. Some infusion reactions such as fever, fatigue, and conjunctivitis were observed in cohorts #1 and #2 (120), but they resolved promptly without medication. Premedication (dexamethasone, acetaminophen, and famotidine) was introduced for the safety of subjects of cohort #3. No adverse event was observed in the first two subjects. The third subject showed local rash with wheal, which resolved soon without medication after cessation of infusion. Because the coronavirus pandemic affected project rescheduling and because the investigational agent expired, cohort #3 was terminated. No problematic clinical sign was observed from any hematological or biochemical analyte, vital sign, electrocardiograph, etc. Blood specimens of cohort #3 provided concentration profiles of HbV in plasma and revealed that HbV circulate in the bloodstream with half-life of approximately 8 h. The circulation half-life of HbV is known dose-dependent, and it will become longer at a practically higher dosage. Based on the results, we are planning the next clinical trial starting from a 100 ml dose. For that purpose, additional GLP preclinical studies, including developmental toxicity studies, are underway in the ongoing project.

## Conclusion

Since the first report of the liposome encapsulated Hb, 45 years have passed. After numerous trials of various lipid compositions and preparation methods, we have ascertained the optimal lipid composition and GMP production method of HbV, and have completed its GLP preclinical toxicological studies and the phase 1 clinical study. Based on the results, we are planning the next clinical trial starting from a 100 ml dose. As shown in Table 2, HbV is useful not only as a transfusion alternative and as an O_2_ carrier, but also as a CO carrier and an antidote for poisoning, which were not expected at the beginning of R&D of HBOCs. Such vast availability to various clinical situations is expected to enhance the contributions of HbV to future human health and welfare. Both CO-HbV and met-HbV are the derivatives of O_2_-HbV but different agents. Their preclinical safety and efficacy studies are the target of our research before starting clinical studies. Other HBOCs are expected to have some potential for use in the same manner.
